# A randomized trial of MONOFIX^®^ vs. V-loc™ for resection bed suture during robotic partial nephrectomy

**DOI:** 10.1186/s12885-024-13213-6

**Published:** 2024-11-27

**Authors:** Jang Hee Han, Gyoohwan Jung, Jung Kwon Kim, Seok-Soo Byun, Seong II Seo, Sung-Hoo Hong, Cheol Kwak, Chang Wook Jeong

**Affiliations:** 1 Department of Urology, Seoul National University Hospital, Seoul National University College of Medicine, Seoul, Korea; 2 Department of Urology, Hanyang University Seoul Hospital, Hanyang University College of Medicine, Seoul, Korea; 3 Department of Urology, Seoul National University Bundang Hospital, Seoul National University College of Medicine, Seongnam, Korea; 4 Department of Urology, Samsung Medical Center, Sungkyunkwan University School of Medicine, Seoul, Korea; 5 Department of Urology, Seoul St. Mary’s Hospital, College of Medicine, The Catholic University of Korea, Seoul, Korea; 6 Department of Urology, Asan Medical Center, Ulsan University College of Medicine, Seoul, Korea

**Keywords:** Barbed suture, Partial nephrectomy, Bed suture time, Monofix, V-Loc

## Abstract

**Background:**

To evaluate the clinical efficacy and safety of Monofix^®^-PDO compared to V-Loc™ for tumor bed suturing during robotic-assisted laparoscopic partial nephrectomy (RAPN).

**Methods:**

A randomized, controlled, multicenter, single-blinded trial was conducted across four tertiary institutions. Patients with T1-2 stage renal masses scheduled for RAPN were enrolled. The exclusion criteria included patients not deemed in need of bed suturing, those with a history of prior chemotherapy or immunotherapy, and those with severe systemic diseases or high bleeding tendencies. A total of 174 patients participated and were subjected to permuted block randomization (T1a vs. others), resulting in 88 patients in the V-Loc™ group and 86 in the Monofix^®^-PDO group. The primary outcome was the resection bed suture time. The secondary outcomes were total suture use time, warm ischemia time, console time (for efficacy), estimated blood loss, hemoglobin change, and 90-day treatment-related adverse events (for safety). All patients were scheduled for follow-up visits for up to three months postoperatively.

**Results:**

The primary outcome, resection bed suture time, did not significantly differ between the V-Loc™ and Monofix^®^-PDO groups (4.8 ± 2.6 vs. 4.5 ± 2.6 min, *p* = 0.531). Secondary outcomes, including total suture used time (5.3 ± 2.8 vs. 4.8 ± 2.6 min, *p* = 0.289) and warm ischemic time (15.6 ± 5.5 vs. 15.4 ± 5.4 min, *p* = 0.834), were comparable between the two groups. In terms of safety outcomes, changes in serum hemoglobin levels did not show significant differences on postoperative days 1, 3, and 14 (*P* = 0.537, 0.353, and 0.840, respectively). No device-related adverse events were observed during the 90-day follow-up period in either group.

**Conclusions:**

Monofix^®^-PDO demonstrated non-inferior to V-Loc in terms of both safety and efficacy in patients undergoing RAPN. This trial is registered on cris.nih.go.kr as KCT0006809 (Registration date: 02/19/2021).

**Supplementary Information:**

The online version contains supplementary material available at 10.1186/s12885-024-13213-6.

## Introduction

Minimally invasive nephron-sparing surgery (NSS) has become the preferred approach for managing small renal tumors due to its equivalent oncologic outcomes and superior functional and survival benefits compared to radical nephrectomy [1]. The advent of robot-assisted partial nephrectomy (RAPN) has further expanded the adoption of NSS, enabling precise tumor excision and effective renorrhaphy techniques [2, 3]. A significant advancement in this regard has been the utilization of barbed sutures (such as V-loc™ and Stratafix™) designed with barbs to self-retain and prevent slipping in the opposite direction. These sutures have shown substantial advantages, including reduced operation time, shorter warm ischemia time, and decreased blood loss [1, 4]. 

While V-Loc™ is currently the most widely used barbed suture, it has certain limitations, such as the absence of a stopper and barbs at the suture’s end and high cost [5], which can hinder its convenience in certain surgical procedures. Therefore, our objective was to identify a suture that could overcome these limitations while remaining competitive with V-loc™, thus expanding the range of suture options available for RAPN. Monofix^®^-PDO, a recent development by Samyang Biopharm in Korea, addresses these issues [6]. Composed of a polydioxanone (PDO) polymer, Monofix^®^-PDO features unidirectional barbs along each strand and includes a stopper, eliminating the need for a separate loop for fixation or tie knots. It also offers a shorter barb-free zone, improved penetration efficiency, greater bending resistance, enhanced tissue-holding strength, and stronger suture durability. In this prospective, single-blinded, randomized controlled trial, we aimed to demonstrate the safety and efficacy of Monofix^®^-PDO as a suture material. Our primary goal was to establish that the effectiveness of the new Monofix^®^-PDO suture does not fall below a pre-defined non-inferiority margin when compared to V-Loc™ in patients undergoing RAPN. Thus, this study seeks to present Monofix^®^-PDO as a promising alternative suture material for RAPN.

## Materials and methods

### Trial design and participants

This multicenter, prospective, randomized controlled trial (RCT) was conducted across four major tertiary centers in the Republic of Korea. Eligible patients, aged 19–85, met the following criteria: (i) clinically diagnosed renal cell carcinoma (RCC), (ii) planned Robot-assisted Partial Nephrectomy (RAPN), (iii) Eastern Cooperative Oncology Group performance status of 0 or 1, and (iv) ability to sign the informed consent form. Exclusion criteria included prior or planned medical oncological treatment; no need for tumor bed suture after tumor resection; moderate to severe cardiovascular, cerebrovascular, respiratory, or liver disease; uncontrolled infections; uncontrolled bleeding tendencies; or refusal to participate. Patient randomization was required within 12 weeks of diagnosis.

The trial protocol was approved by the Institutional Review Board of Seoul National University Hospital, Seoul, Republic of Korea (2007-008-1140). Written informed consent was obtained from all patients. The study was performed in accordance with the applicable laws and regulations, good clinical practice, and ethical principles as described in the Declaration of Helsinki. The study adhered to CONSORT guideline and the results are reported as per the CONSORT statement.

### Randomization

Central randomization was performed by the Medical Research Collaborating Center (MRCC) team at Seoul National University Hospital using a web-based system. Stratification was based on the clinical T stage, categorized as T1a points or others. Subjects were randomly assigned in a 1:1 ratio to either the Monofix^®^-PDO or V-Loc™ group. A randomly permuted block design with varying block sizes was employed to develop a random allocation list for each stratum, with separate lists generated for each participating institution. The research participants and their guardians were blinded to the random allocation, and the blinding procedure was rigorously maintained throughout the study.

### Intervention

According to the random assignment, the experimental (Monofix^®^-PDO) and the control groups (V-loc™) performed RAPN using designated sutures for hemostasis at the tumor bed resection site. During surgery, the first or second assistant participating in the procedure requested the surgical room nurse to (1) measure the time taken for each suture product to be prepared from opening the packaging to being ready for use in the actual surgery for each opened suture, such as attaching additional items, such as Hem-o-lok^®^, Lapra-ty^®^, or extra tie knots (suture preparation time) and (2) measure the time taken for hemostasis at the tumor resection bed. Each duration was recorded in the surgical records along with surgical findings.

### Data collection

Study data were collected online using the Research Electronic Data Capture (REDCap) program hosted by the Department of Urology at Seoul National University Hospital. REDCap is a secure, web-based application that supports data capture for research studies [7]. At baseline, patient demographics were registered: performance status, social/medical history, comorbidity, kidney cancer-related clinical information, operation finding, and Charlson Comorbidity Index. Laboratory tests, postoperative outcomes, and adverse events were recorded during follow-up. Adverse events were classified according to the Clavien-Dindo grading system.

### Primary and secondary study outcomes

The primary outcome of this trial was the resection bed suture time. The secondary outcomes were total suture usage time (suture preparation time + resection bed suture time), warm ischemia time, console time, and safety outcomes such as 90-days treatment-related adverse events, postoperative complications, estimated blood loss, and differences in hemoglobin levels before and after surgery.

### Statistical analyses

This was a non-inferiority study. In a prospective randomized study of laparoscopic partial nephrectomy using V-loc™, the bed suture time was 10.4 ± 3.7 min, whereas conventional sutures (non-barbed suture) took 13.8 ± 5.6 min (*p* = 0.01) [8]. Based on this, we assumed that if the bed suture time for Monofix^®^-PDO is shorter than 12.7 min, the median bed suture time of the two groups (10.4 min and 13.8 min) in the mentioned study, then it can be considered non-inferior. In other words, the non-inferiority margin was calculated as 1.7 min. Assuming a power of 90% and a two-sided significance level of 5%, each group required 82 subjects for a total of 164 subjects. Assuming a dropout rate of 5%, 174 patients were required. Statistical comparisons were performed using the chi-squared test (for categorical variables) and t-test (for continuous variables). Statistical significance was set at p values < 0.05.

## Results

### Patients characteristics

**Fig. 1:**
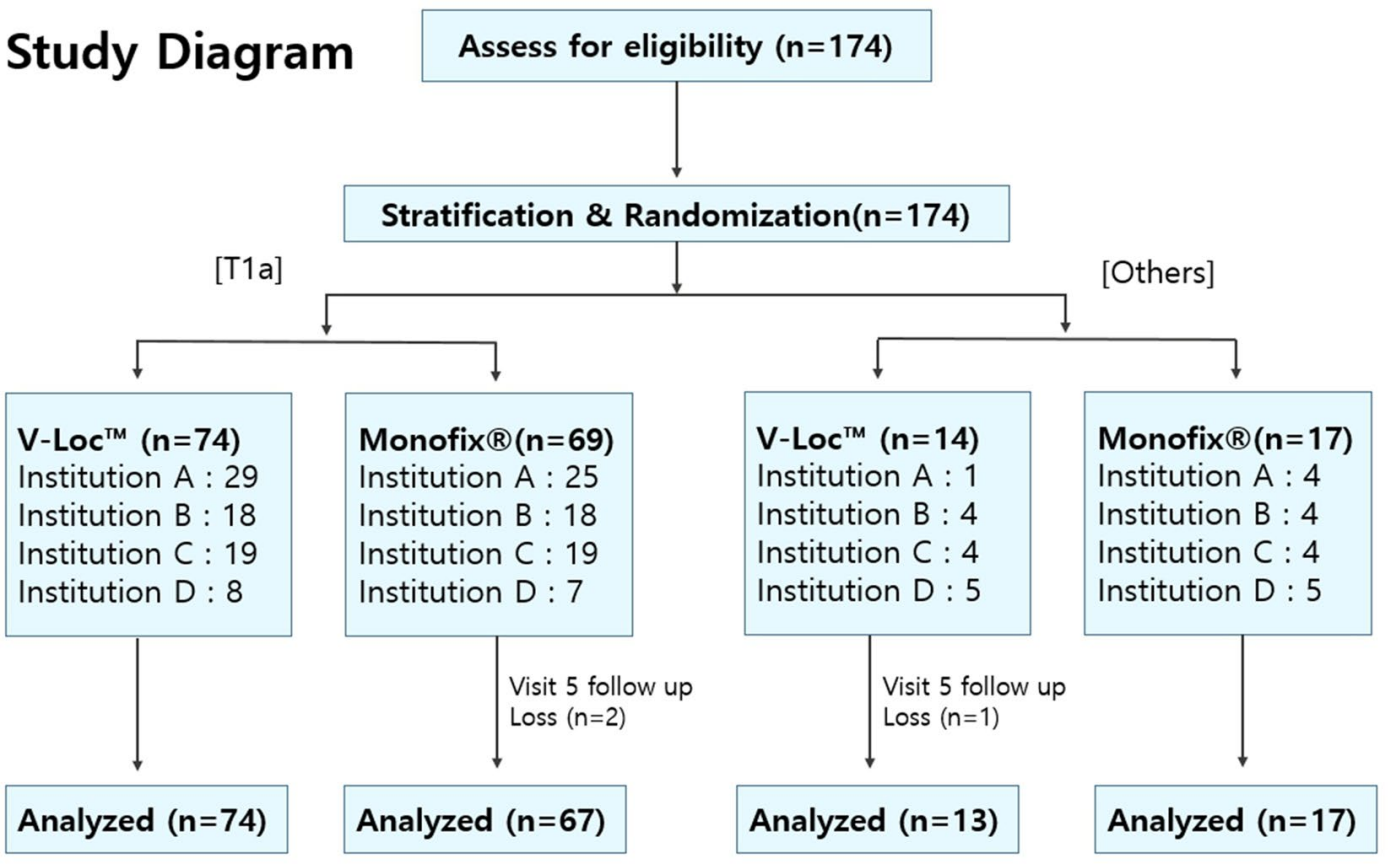
CONSORT flow diagram and randomization

Between May 2021 and March 2022, 174 eligible patients were enrolled across three tertiary centers. We analyzed the intent-to-treat group of patients (*n* = 174), including three patients who could not reach their last follow-up visit (three months postoperative). The number of patients with T1a stage disease was 143 (81.7%), whereas 31 patients had T1b stage disease (17.7%). In terms of tumor complexity, 93 patients (53.4%) harbored low-complexity tumors, whereas 81 patients (46.6%) harbored moderate-to-high-complexity tumors (Supplementary Table 1). Overall, 88 patients were allocated to the V-loc™ group, while 86 patients were assigned to the Monofix^®^-PDO group (Fig. 1). The clinical characteristics of the treatment and control groups were well-balanced (Table 1).

**Table 1:** Baseline characteristics of the V-Loc™ and Monofix^®^ groups

	V-Loc™ (*n* = 88)	MONOFIX^®^ (*n* = 86)	*p*-value
Age (years)	53.2 ± 12.2	54.5 ± 13.4	0.483
BMI (kg/m2)	25.4 ± 3.9	24.7 ± 3.0	0.180
Sex (Male) (n, %)	50 (56.8)	60 (69.8)	0.077
HTN (n, %)	39 (44.3)	37 (43.0)	0.863
DM (n, %)	8 (9.1)	11 (12.8)	0.434
Clinical T stage (n, %)			0.641
T1a	74 (84.1)	69 (80.2)	
T1b	14 (15.9)	17 (19.8)	
Tumor exophytic/endophytic (n, %)			0.939
Exophytic mass	71 (80.7)	68 (79.1)	
Entirely Endophytic mass	17 (19.3)	18 (20.9)	
Tumor size (cm)	2.65 ± 1.11	2.53 ± 1.19	0.479
R.E.N.A.L score	6.42 ± 2.08	6.71 ± 1.88	0.339
Pathology (n, %)			0.448
Clear cell RCC	60 (68.2)	65 (75.6)	
Non-clear cell RCC	16 (18.2)	10(11.6)	
Benign pathology	12 (13.6)	11 (12.8)	
Creatinine (mg/dL)	0.84 ± 0.20	0.85 ± 0.19	0.725
MDRD eGFR (mL/min/1.73 m²)	90.5 ± 16.3	91.3 ± 17.4	0.764

BMI, body mass index; HTN, hypertension; DM, diabetes mellitus; RCC, renal cell carcinoma; MDRD, Modification of Diet in Renal Disease Study equation; eGFR, Estimated Glomerular Filtration Rate

As shown in Table 2, non-barbed sutures were not used during the resection bed suturing, and the number of barbed suture materials used was also comparable between the treatment and control groups.

**Table 2:** Comparison of primary and secondary outcomes between V-Loc™ and MONOFIX^®^

	V-Loc™ (*n* = 88)	Monofix^®^ (*n* = 86)	*p*-value
No. of used barbed suture material			0.585
1	46 (52.3)	44 (51.2)	
2	30 (34.1)	34 (39.5)	
3–4	12 (13.7)	8 (9.3)	
Barbed suture preparation time (sec)^¶^	24 [15.3, 41.0]	0 [0, 16]	< 0.001
Resection bed suture time (min)	4.8 ± 2.6	4.5 ± 2.6	0.531
Non-barbed suture time (min)	0	0	1.000
Total bed suture time (min)	5.3 ± 2.8	4.8 ± 2.6	0.289
Warm ischemic time (min)	15.6 ± 5.5	15.4 ± 5.4	0.834
Total console time (min)	54.7 ± 20.4	53.6 ± 18.6	0.694
Operation time (min)	85.3 ± 23.9	85.9 ± 21.2	0.854
Estimated blood loss (cc) ^¶^	50 [50, 100]	50 [50, 100]	0.529
Additional suture after declamping	14 (15.9)	11 (12.8)	0.558
Blood Transfusion	0	0	1.000
Hemoglobin drop ^¶^			
POD#1 – Preop	-1.5 [-0.8, -2.3]	-1.5 [-0.8, -2.1]	0.537
POD#3 – Preop	-2.4 [-1.6, -3.0]	-2.2 [-1.3, -3.0]	0.353
POD#14 – Preop	-0.6 [-0.1, -1.2]	-0.6 [-0.0, -1.3]	0.840
eGFR change % (POD#3m/Preop)	0.97 ± 0.14	0.97 ± 0.15	0.733

POD, postoperative day; eGFR, estimated glomerular filtration rate

^¶^ Values are presented as the median (interquartile range)

### Endpoints

#### Entire cohort

The primary outcome of this trial was the resection bed suture time. The control group took 4.8 ± 2.6 min, while the treatment group took 4.5 ± 2.6 min (*p* = 0.531). Secondary outcomes were analyzed. In terms of total suture usage time (suture preparation time + resection bed suture time), taken time were also comparable between the two groups (5.3 ± 2.8 min (control) vs. 4.8 ± 2.6 min (treatment), *p* = 0.289). Other outcomes, such as warm ischemic time, console time, estimated blood loss, and differences in hemoglobin levels before and after surgery, were comparable between the two groups (Table 2).

#### Sub-cohort (Low complexity group vs. Moderate-high complexity group)

In the low-complexity group, 49 and 44 patients were included in the control and treatment groups, respectively. Resection bed and total bed suture times were compared, which were comparable between the two groups. The other secondary outcomes were also comparable. (Supplementary Table 2).

For the moderate to high complexity group, resection bed suture time for the control group was 6.3 ± 2.8 min and 5.1 ± 3.0 min in the treatment group (*p* = 0.074). Meanwhile, total bed suture time was significantly shorter in the treatment group (5.5 ± 3.1 vs. 6.9 ± 2.8 min, *p* = 0.046). The other secondary outcomes were also comparable.

### Adverse event

In the control group, 23 (26.1%) patients experienced Clavien-Dindo Grade I-II adverse events within 90 days postoperatively, while there were 22 (25.6%) in the treatment group (*p* = 1.000). Clavien-Dindo Grade III or higher adverse events occurred in one (1.1%) patient in the control group and four (4.7%) in the treatment group. No device-related adverse events were observed (Table 3).

**Table 3:** Comparison of adverse events Associated with V-Loc™ and Monofix^®^ according to the Clavien-Dindo Grading System

	V-Loc™ (*n* = 88)	Monofix^®^ (*n* = 86)	*p*-value
Grade I-II (n, %)	23 (26.1)	22 (25.6)	1.00
Device related (n, %)	0 (0.0)	0 (0.0)	1.00
Grade III (n, %)	1 (1.1)	4 (4.7)	0.35
Device related (n, %)	0 (0.0)	0 (0.0)	1.00

## Discussion

In this multicenter RCT, Monofix^®^-PDO was comparable to V-Loc™ in terms of both safety and efficacy in patients undergoing RAPN, meeting both the primary and secondary outcomes. There were also clinical benefits of using Monofix^®^-PDO in patients with moderate to high complexity tumors. Based on the known finding that conventional suture materials are more time-consuming and that the knots may be prone to breakage or extrusion [9], our findings suggest that Monofix^®^-PDO can be considered an effective alternative suture material for RAPN, potentially expanding the utilization of partial nephrectomy, even in cases involving complex renal masses [10]. 

Barbed sutures, especially V-loc™, are now widely utilized in various minimally invasive surgical fields. For example, in a laparoscopic myomectomy RCT comparing barbed versus conventional sutures, they demonstrated significant benefits such as reduced surgery time, decreased bleeding, and improved surgical convenience compared to conventional sutures [11]. Similarly, in a robotic-assisted laparoscopic hysterectomy comparative analysis, when compared to interrupted vicryl sutures, V-loc™ substantially reduced the time required for vaginal cuff closure, with less blood loss and less observed granulation tissue [12]. In the field of general surgery, in obese patients undergoing laparoscopic gastric bypass surgery, these sutures effectively reduced the time required for intestinal anastomosis [13]. In the field of urology, in robot-assisted radical prostatectomy, barbed sutures offered advantages in terms of continence rate, length of catheterization, and other surgical outcomes [14]. Many other urological surgeries have reported the clinical benefits of barbed sutures, such as robot-assisted radical cystectomy [15] and robot-assisted laparoscopic pyeloplasty [16]. 

In this clinical trial, we specifically targeted patients who underwent RAPN because it was hypothesized that using barbed sutures could yield maximal benefits in this context. A systematic review and meta-analysis by Lin et al. included eight cohort studies (one RAPN, five laparoscopic partial nephrectomies, one open partial nephrectomy, and one combination of laparoscopic and open partial nephrectomy) [17] and concluded that barbed sutures significantly reduced the operative time, warm ischemic time, and postoperative complications. The benefits of barbed sutures in partial nephrectomy appear clear; however, no head-to-head comparison studies have been conducted on each suture material. Sammon et al. conducted a prospective study specifically, using V-Loc™ barbed suture in RAPN. Their results highlighted a notable reduction in warm ischemic time with the use of barbed sutures [1]. In the meanwhile, although retrospective data, studies by Shang et al. and Xu et al. reported that using Quill™ barbed sutures led to a low complication rate and significantly shorter warm ischemic time [18, 19]. 

We expected three key benefits from using this new suture material. Firstly, we aimed to prove that the new suture does not compromise bed suture time and warm ischemic time compared to the existing V-Loc™ sutures. Secondly, our goal was to reduce the labor of surgical nurses by reducing suture preparation time and achieve surgical standardization and safety by eliminating the need for additional items like Hem-o-lok^®^, Lapra-ty^®^, or extra tie knots, which are required when using V-Loc™ for bed suturing. Indeed, the time needed for suture material preparation was significantly shorter with Monofix^®^-PDO compared to V-Loc™. Thirdly, the study considered the clinical perspectives discussed by Sammon et al., who reported challenges when using barbed sutures in their RAPN procedures [1]. Notably, V-Loc™ lacked barbs in the first 2 cm, necessitating readjustment and occasionally engaging with tissue, making them challenging to manage. In contrast, the Monofix^®^-PDO utilized in this study had a shorter barb-free zone, which enhanced its usability. We also achieved bed suture time savings from the three clinical perspectives mentioned above in moderate-to-high-complexity tumor cases.

Despite the promising results, this study has several limitations. First, safety outcomes and renal function were presented up to a three-month follow-up period. However, it would be valuable to provide data with an extended follow-up duration, as there are concerns regarding potential inflammation or fibrosis induced by the suture barbs, particularly in the case of urinary tract reconstruction [20]. Also, in terms of renal function, long term data will be needed to confirm the clinical efficacy of this new suture material. This prolonged follow-up data could offer a more comprehensive understanding of the safety profile. Second, the clinical trial employed a stratified design, categorizing patients into the T1a vs. other groups. Notably, the “others” category predominantly comprised T1b tumors, and their representation in the study was relatively small (*n* = 31/174). Therefore, the results pertain primarily to T1a tumors, raising concerns about generalizing these findings to tumors of all sizes in the context of RAPN. Additionally, for the T1a tumors, while the sample size was sufficient to support our findings, having fewer than 100 patients in each group may limit the generalizability of our results. Third, a distinctive feature of this study was its exclusive focus on barbed sutures for bed suturing, i.e., the inner layer of the renorrhaphy. Consequently, a pre-defined standardization of the outer layer renorrhaphy technique across institutions and surgeons is lacking. Furthermore. In a similar context, tumors initially considered sutureless were excluded, which could be seen as a limitation, as this decision was based on the individual judgment of surgeons at each institution. However, this effect was likely minimized through stratification by institution during the randomization process. It is noteworthy that despite this limitation, there were no significant differences in warm ischemic time or bed suture time between the control and treatment groups. This suggests that the findings of this study may have mitigated the clinical impact of this limitation. These limitations should be considered when interpreting the results and considering the broader applicability of Monofix^®^-PDO sutures in RAPN procedures. Future research efforts could focus on evaluating the long-term outcomes and conducting cost-effectiveness analyses of Monofix^®^-PDO and V-Loc™ suturing techniques in a larger and more diverse patient population. Additionally, comparative studies incorporating advanced imaging techniques and functional outcomes assessments could further clarify the optimal choice of suturing materials in renal mass surgery. Additionally, this study was conducted on a group that underwent double-layer renorrhaphy; however, since systemic review and meta-analysis have reported that single-layer renorrhaphy is superior in terms of renal function preservation [21], it would be beneficial to conduct a comparative analysis with a single-layer renorrhaphy group in future research.

## Conclusions

In this multicenter prospective RCT, Monofix^®^-PDO demonstrated safety and efficacy comparable to those of V-Loc™ in patients undergoing RALPN. These findings suggest that the introduction of Monofix^®^-PDO can be considered in a wider spectrum of surgical procedures in which barbed sutures are employed.

## Electronic supplementary material

Below is the link to the electronic supplementary material.

Supplementary Material 1

Supplementary Material 2

## Data Availability

No datasets were generated or analysed during the current study.

## Abbreviations

RAPN: Robot-assisted partial nephrectomy
NSS: Nephron-sparing surgery
RCT: Randomized controlled trial
RCC: Renal cell carcinoma
