# Supplementary material for: A randomized trial of MONOFIX® vs. V-loc™ for resection bed suture during robotic partial nephrectomy
Source: BMC Cancer. 2024 Nov 27;24:1458. doi: 10.1186/s12885-024-13213-6 (PMC11600744; doi:10.1186/s12885-024-13213-6)
Supplement: Supplementary file 1 — Supplementary Material 1 [file 12885_2024_13213_MOESM1_ESM.docx]

**Supplementary Table 1. Baseline characteristics**

| Total (n) | 174 |
| --- | --- |
| Age (years) | 53.8±12.8 |
| BMI (kg/m2) | 25.1±3.5 |
| Sex (Male) (n, %) | 110 (62.9) |
| HTN (n, %) | 76 (43.4) |
| DM (n, %) | 19 (10.9) |
| Clinical T stage (n, %) |  |
| T1a | 143 (81.7) |
| T1b | 31 (17.7) |
| Tumor exophytic/endophytic (n, %) |  |
| Exophytic mass | 139 |
| Entirely Endophytic mass | 35 |
| Tumor size (cm) | 2.6±1.1 |
| R.E.N.A.L score | 6.6±2.0 |
| Low | 93 (53.4) |
| Moderate to High | 81 (46.6) |
| Pathology (n, %) |  |
| Clear cell RCC | 125 |
| Non-clear cell RCC | 36 |
| Benign pathology | 23 |

BMI, body mass index; HTN, hypertension; DM, diabetes mellitus; RCC, renal cell carcinoma.
