# Supplementary material for: A randomized trial of MONOFIX® vs. V-loc™ for resection bed suture during robotic partial nephrectomy
Source: BMC Cancer. 2024 Nov 27;24:1458. doi: 10.1186/s12885-024-13213-6 (PMC11600744; doi:10.1186/s12885-024-13213-6)
Supplement: Supplementary file 2 — Supplementary Material 2 [file 12885_2024_13213_MOESM2_ESM.docx]

**Supplementary Table 2. Comparison of primary and secondary outcomes between V-Loc™ and MONOFIX® in Low and Moderate-High complexity groups.**

|  | **V-Loc™**  **(n=88)** | **Monofix®**  **(n=86)** | p-value |
| --- | --- | --- | --- |
| Low complexity group | 49 (55.7) | 44 (51.2) | 0.550 |
| Resection bed suture time (min) | 3.6±1.9 | 4.0±2.0 | 0.353 |
| Total bed suture time (min) | 4.0±2.0 | 4.15±2.0 | 0.690 |
| Warm ischemic time (min) | 12.5±4.0 | 13.0±3.6 | 0.577 |
| Estimated blood loss (cc) | 90±96 | 69±37 | 0.156 |
| Hemoglobin drop |  |  |  |
| POD#1 – Preop | -1.6±1.1 | -1.2±0.9 | 0.065 |
| POD#3 – Preop | -2.2±1.4 | -1.7±1.1 | 0.075 |
| POD#14 – Preop | -0.7±0.9 | -0.6±1.7 | 0.725 |
|  |  |  |  |
| Moderate-High complexity group | 39 (44.3) | 42 (48.8) | 0.550 |
| Resection bed suture time (min) | 6.3±2.8 | 5.1±3.0 | 0.074 |
| Total bed suture time (min) | 6.9±2.8 | 5.5±3.1 | 0.046 |
| Warm ischemic time (min) | 19.4±4.8 | 17.9±5.8 | 0.226 |
| Estimated blood loss (cc) | 118±111 | 103±89 | 0.507 |
| Hemoglobin drop |  |  |  |
| POD#1 – Preop | -1.6±0.9 | -1.7±0.9 | 0.505 |
| POD#3 – Preop | -2.7±1.0 | -2.7±1.3 | 0.733 |
| POD#14 – Preop | -0.8±1.2 | -1.0±1.0 | 0.608 |

POD, postoperative day.
